# Updated guidelines for chronic active Epstein–Barr virus disease

**DOI:** 10.1007/s12185-023-03660-5

**Published:** 2023-09-20

**Authors:** Jun-ichi Kawada, Yoshinori Ito, Koichi Ohshima, Masaki Yamada, Shinsuke Kataoka, Hideki Muramatsu, Akihisa Sawada, Taizo Wada, Ken-Ichi Imadome, Ayako Arai, Keiji Iwatsuki, Shouichi Ohga, Hiroshi Kimura, The Committee for Guidelines for the Management of Chronic Active EBV Disease, Related Disorders (the MHLW Research Team in Japan)

**Affiliations:** 1 Department of Pediatrics, Nagoya University Graduate School of Medicine, Nagoya, Japan; 2 Department of Pediatrics and Child Health, Nihon University School of Medicine, Tokyo, Japan; 3 Department of Pathology, School of Medicine, Kurume University, Kurume, Japan; 4 Department of Advanced Medicine for Viral Infections, National Center for Child Health and Development, Tokyo, Japan; 5 Department of Hematology/Oncology, Osaka Women’s and Children’s Hospital, Izumi, Japan; 6 Department of Pediatrics, Kanazawa University, Kanazawa, Japan; 7 Division of Hematology and Oncology, Department of Internal Medicine, St. Marianna University School of Medicine, Kawasaki, Japan; 8 Department of Dermatology, Dentistry, and Pharmaceutical Sciences, Okayama University Graduate School of Medicine, Okayama, Japan; 9 Department of Pediatrics, Graduate School of Medical Sciences, Kyushu University, Fukuoka, Japan; 10 Department of Virology, Nagoya University Graduate School of Medicine, Nagoya, Japan

**Keywords:** EBV, CAEBV, Diagnostic criteria, Guidelines

## Abstract

Chronic active Epstein–Barr virus disease (CAEBV), formerly named chronic active Epstein–Barr virus infection, is characterized by systemic inflammation and clonal proliferation of Epstein–Barr virus (EBV)-infected T or NK cells. As CAEBV is a potentially life-threatening illness, appropriate diagnosis and therapeutic interventions are necessary for favorable clinical outcomes. Substantial evidence regarding the pathogenesis and treatment of CAEBV has been accumulated since previous guidelines for the diagnosis of CAEBV were proposed. To reflect this evidence, we updated the guidelines for the diagnosis and treatment of CAEBV to improve clinical management of the disease. The details of the updated guidelines are presented in this report. Diagnosis of CAEBV now requires confirmation of a high copy number of EBV genome and EBV-infected T or NK cells. An EBV DNA load ≥ 10,000 IU/mL in whole blood is proposed as the diagnostic cutoff value for CAEBV in this updated guideline. A standard treatment approach for CAEBV has not been established, and hematopoietic stem cell transplantation (HSCT) is considered the only curative treatment. Chemotherapy can be administered to control disease activity before HSCT.

## Introduction

Epstein–Barr virus (EBV) is a ubiquitous virus that infects more than 90% of adults worldwide. EBV infection is typically asymptomatic; however, a delayed primary infection among adolescents and young adults has been linked to the symptomatic condition known as infectious mononucleosis. EBV can cause chronic illness characterized by prolonged infectious mononucleosis-like symptoms and sustained high EBV DNA load in peripheral blood. This condition is known as “chronic active EBV disease” or “chronic active EBV infection,” both abbreviated as CAEBV (ICD-O: 9725/1) [1, 2]. In this article, CAEBV is used as an abbreviation for chronic active EBV disease. Some patients with CAEBV initially develop cutaneous symptoms of hydroa vacciniforme lymphoproliferative disorder (HV-LPD) or severe mosquito bite allergy (SMBA), and then progress to more serious conditions that fulfill the diagnostic criteria for CAEBV [3]. Conversely, it is not rare for CAEBV patients to present with skin rash similar to HV-LPD and SMBA during the clinical course.

In East Asian countries, including Japan, CAEBV is characterized by systemic inflammation accompanied by clonal proliferation of EBV-infected T or NK (T/NK) cells. In the “5th Edition of the World Health Organization (WHO) Classification of Hematolymphoid Tumors” revised in 2022, CAEBV is listed as one of the EBV-positive T- and NK-cell lymphoid proliferations and lymphomas of childhood and is named “systemic chronic active EBV disease” [4, 5]. Furthermore, the disease name “CAEBV” used in the present article includes HV-LPD and SMBA with persistent systemic symptoms and/or organ disease. In the International Consensus Classification of Mature Lymphoid Neoplasms, CAEBV is described as a mature T- and NK-cell neoplasm and is named “chronic active Epstein–Barr virus disease, systemic (T cell and NK cell phenotype)” [6].

CAEBV is a potentially life-threatening illness; thus, it is necessary to establish appropriate diagnostic methods and treatment strategies to facilitate optimal clinical management of the disease. In 2005, the Japanese Association for Research on EBV and Related Diseases published the article “Proposed Guidelines for Diagnosing Chronic Active Epstein–Barr Virus Infection” [7]. Since then, considerable progress has been made in the standardization of diagnostic procedures for CAEBV. In addition, substantial evidence regarding the pathogenesis and treatment of CAEBV has been accumulated. Considering these advancements in CAEBV research, we updated the guidelines for the diagnosis and treatment of CAEBV for improved clinical management of the disease. The details of the updated guidelines are presented in this report.

## Etiology

The pathogenesis of CAEBV is not yet fully understood. CD21 and HLA-DR are considered EBV receptors in B cells; however, the receptors in T/NK cells have not been identified. Therefore, little is known about the mechanism underlying EBV infection in T/NK cells. CAEBV develops in the apparent absence of immunodeficiency. However, EBV-infected T/NK cells are not removed by cytotoxic T cells. Previous studies have demonstrated that patients with CAEBV show impaired T/NK-cell activity against EBV-infected cells [8, 9]. It is possible that T/NK-cell activity is secondarily suppressed by the development of disease conditions in CAEBV.

How EBV induces T/NK-cell proliferation remains unclear. Previous studies have demonstrated the activation of several pathways, such as the NF-κB, mTOR, and JAK/STAT3 pathways, in EBV-positive T/NK-cell lines or T/NK cells from patients with CAEBV [10–13]. EBV may contribute to the lymphomagenesis of T/NK cells through the activation of these pathways, which could be considered new therapeutic targets.

A recent comprehensive genetic analysis revealed that somatic driver mutations, including *DDX3X* and other genes related to malignancies, are frequently found in EBV-infected T/NK cells of patients with CAEBV [14]. The clonal evolution of EBV-infected cells compromising multiple cell lineages was also confirmed in that study. Notably, *DDX3X* mutations are observed in an EBV-related NK/T-cell lymphoma [15]. Therefore, lymphomagenesis in CAEBV could be related to serial acquisition of somatic mutations in T/NK cells. In the above-mentioned comprehensive genetic analysis, frequent intragenic deletions that affected *Bam*HI A rightward transcript microRNA clusters and several EBV genes were detected in patients with CAEBV [14]. These intragenic deletions were also frequently detected in patients with various EBV-associated neoplastic disorders; however, they were not detected in patients with infectious mononucleosis. These deletions, which are related to upregulation of the lytic cycle, led to the enhancement of lymphomagenesis in a xenograft model [14, 16]. Therefore, lytic cycle-associated EBV genes may be involved in lymphomagenesis in EBV-associated neoplastic disorders, including CAEBV [17, 18].

## Epidemiology

CAEBV is most common in East Asia; however, it has been documented in Mexico and, occasionally, in Western countries. The reason for this distribution pattern is unknown; however, the genetic propensity of individuals with CAEBV has been speculated. A previous report has shown a positive correlation between CAEBV and human leukocyte antigen (HLA) A26, which is frequently detected in East Asians [19].

CAEBV was first identified as a childhood disease. However, as more physicians are becoming aware of the disease, more cases of CAEBV in adults are being documented. The mean age of onset of 82 patients with CAEBV included in a Japanese nationwide survey was 11.3 years [20]. However, the median age of 100 patients included in a recent survey, which was conducted from 2016 to 2018, was 21 years, and the number of adult (≥ 20 years) patients was greater than the number of pediatric patients [21]. In these surveys, the prognosis of older patients (age of onset, ≥ 8 years) with CAEBV or adult-onset CAEBV was worse than that of younger patients [20, 21].

## Clinical features

CAEBV is characterized by persistent infectious mononucleosis-like symptoms, including fever, swollen lymph nodes, and hepatosplenomegaly. Patients with CAEBV often develop systemic organ diseases, such as hemophagocytic lymphohistiocytosis (HLH), peptic ulcer, intestinal pneumoniae, vasculitis, uveitis, liver failure, and coronary artery aneurysms. Although some patients experience serious symptoms that worsen rapidly, others remain stable without treatment.

HV-LPD and SMBA are cutaneous manifestations occasionally observed in patients with CAEBV. HV-LPD is a photosensitivity dermatosis characterized by vesicular lesions on sun-exposed skin that eventually heal, leaving depressed scars [3]. SMBA is a cutaneous reaction characterized by swelling, edematous skin reactions, and deep ulcers at the sites of bug or mosquito bites and even vaccination injections. HV-LPD and SMBA are considered cutaneous variants of EBV-positive T/NK lymphoproliferative disorders and are listed as independent diseases in the WHO Classification of Hematolymphoid Tumors [4]. However, both diseases may overlap and progress to more serious conditions. Therefore, CAEBV should be considered if patients with HV-LPD and SMBA present with persistent systemic symptoms and/or organ disease.

Throughout the course of CAEBV, hematological malignancies, such as aggressive NK-cell leukemia and extranodal NK/T-cell lymphoma, nasal type, occasionally develop [20, 22]. These malignancies can take many months to several decades from disease onset to develop.

## Diagnosis

Diagnosis of CAEBV is based on a combination of clinical features and results of virological studies. The diagnostic criteria for “severe, chronic EBV infection” and “severe chronic active EBV infection syndrome” were independently proposed in 1988 and 1991, respectively [23, 24]. These criteria consist of presentation of persistent infectious mononucleosis-like symptoms, hematological abnormality/major organ involvement, and abnormal EBV antibody titers. In the 2005 Proposed Guidelines for Diagnosing Chronic Active Epstein–Barr Virus Infection published by the Japanese Association for Research on EBV and Related Diseases, quantitative EBV polymerase chain reaction (PCR) test was recommended as a specific laboratory test for diagnosing CAEBV [7].

In 2022, the Ministry of Health Labor and Welfare research team in Japan revised the diagnostic criteria for CAEBV (Table 1). In these revised diagnostic criteria, confirmation of elevated number of EBV genomes and EBV-infected T/NK cells are required for the diagnosis of CAEBV because the available evidence suggests that CAEBV is associated with the proliferation of EBV-infected T/NK cells. Details of laboratory tests for diagnosing CAEBV are described as follows.

**Table 1:** Proposed diagnostic criteria for CAEBV

1. Persistent or recurrent infectious mononucleosis-like symptoms for more than 3 months
2. Detection of an increased number of EBV genomes in peripheral blood and/or affected tissues
3. Detection of EBV-infected T or NK cells in peripheral blood and/or affected tissues
4. Chronic illness that cannot be explained by other known disease processes at the time of diagnosis

All four criteria should be met before CAEBV can be diagnosed
Notes
1. Infectious mononucleosis-like symptoms include fever, swollen lymph nodes, and hepatosplenomegaly. Additional complications include hematological, digestive tract, neurological, pulmonary, ocular, dermal (hydroa vacciniforme lymphoproliferative disorder and severe mosquito bite allergy), and cardiovascular (aneurysm and valvular disease) disorders. Hemophagocytic lymphohistiocytosis due to primary EBV infection is not considered CAEBV. Hydroa vacciniforme lymphoproliferative disorder and severe mosquito bite allergy with persistent systemic symptoms and/or organ disease are considered CAEBV. Patients with CAEBV often develop hemophagocytic lymphohistiocytosis, T/NK lymphoma, or leukemia during the course of the disease; however, the diagnosis of CAEBV as a primary illness remains
2. More than 10,000 IU/mL (4.0 log IU/mL) of EBV DNA is detected in whole-blood samples using real-time polymerase chain reaction. EBER in situ hybridization is performed for the detection of EBV-positive cells in a tissue
3. Primary or acquired immunodeficiencies, rheumatic diseases, malignant lymphoma (Hodgkin lymphoma, ENKL, angioimmunoblastic T-cell lymphoma, and peripheral T-cell lymphoma, not otherwise specific), aggressive NK-cell leukemia, and iatrogenic immunodeficiency should be differentiated from CAEBV. The following is a list of suggested laboratory tests for investigating the type of CAEBV and making a differential diagnosis:
(a) EBV-related antibody
High EBV-related antibody titers measured using fluorescence antibody tests (VCA-IgG ≥ 640 and EA-IgG ≥ 160) are unique virological findings of CAEBV. VCA- and/or EA-IgA antibodies are often detected. High EBV-related antibody titers are not necessary for the diagnosis of CAEBV because some patients do not exhibit high antibody titers
(b) Clonality of EBV-infected cells
I. Southern blot hybridization with a probe targeting the EBV terminal repeats
II. *TCR* gene rearrangement
(c) Histopathological and molecular evaluation
I. General histopathology
II. Immunohistological staining
III. Chromosomal analysis
IV. Rearrangement studies (e.g., immunoglobulin, TCR)
(d) Immunological studies
I. Marker analysis of peripheral blood (including HLA-DR)
II. General immunological studies (immunoglobulin levels, complement levels, T-cell function proliferation assays, NK-cell cytotoxicity, neutrophil function tests)
III. Cytokine analysis

*EBV* Epstein–Barr virus, *CAEBV* chronic active EBV disease, *EBER* EBV-encoded RNA, *ENKL* extra nodal NK/T-cell lymphoma nasal type, *VCA* viral capsid antigen, *EA* early antigen, *TCR* T-cell receptor, *HLA* human leukocyte antigen

### Virological studies

#### EBV-related antibodies

Unusual EBV antibody profiles, such as extremely high anti-viral capsid antigen (VCA)-IgG (≥ 640) and anti-diffuse and restricted early antigen (EA-DR)-IgG (≥ 160) titers measured using fluorescence antibody tests are considered virological laboratory findings of CAEBV and have been included in the previous diagnostic criteria [23–25]. However, EBV-seropositive healthy individuals can have a high VCA-IgG level without any disease. Moreover, not all patients with CAEBV show high EBV-related antibody titers. The geometric mean titer for the VCA-IgG of T-cell CAEBV is 2010, whereas that of NK-cell CAEBV is 310. Furthermore, the geometric mean titers for the anti-EA-DR-IgG of T-cell and NK-cell CAEBV are 610 and 70, respectively [26]. An unusual pattern of EBV-related antibodies is frequently observed in T-cell CAEBV. However, it should be noted that some patients with CAEBV, particularly those with NK-cell CAEBV, do not show high EBV-related antibody titers. Therefore, it is considered that high antibody titers are not necessary for the diagnosis of CAEBV.

#### EBV DNA quantification

Real-time PCR measurement of EBV DNA load is commonly performed for the diagnosis or monitoring of EBV-associated diseases [27]. Patients with CAEBV often show extremely high EBV DNA loads in peripheral blood [28, 29]. Real-time PCR can be used to assess EBV DNA load in whole blood or other peripheral blood components, such as peripheral blood mononuclear cells (PBMCs) and plasma. PBMCs and plasma contain cell-associated and cell-free EBV DNA, respectively. Therefore, disease status or etiology should be considered when determining the best blood component to be assessed for diagnosing each EBV-associated disease.

EBV DNA load increases in all blood components during the active phase of CAEBV, whereas plasma/serum EBV DNA is occasionally undetectable during the inactive phase [22, 28]. Therefore, EBV DNA load testing in whole blood or PBMCs is preferred for the diagnosis of CAEBV. Conversely, it has been demonstrated that patients with CAEBV in the active phase have higher plasma EBV DNA loads than those with inactive disease [29]. In a recent study by Zhen et al., EBV DNA load in plasma was considerably more accurate than that in PBMCs in differentiating disease activity in NK-cell-type CAEBV [30]. Therefore, plasma EBV DNA load is more strongly associated with the disease activity in CAEBV than EBV DNA load in PBMCs or whole blood.

The threshold values of EBV DNA load for diagnosing CAEBV have not been established. In 2016, the WHO International Standard for EBV DNA was released to standardize quantitative PCR and to represent EBV DNA loads with an international unit (IU) [31]. Although real-time PCR assay for EBV has not been standardized completely, comparison of EBV DNA loads across institutions has become easier with this standard. The authors of a previous study showed that the whole-blood EBV DNA loads of 29 (94%) of 31 patients with CAEBV were higher than 10,000 IU/mL, and EBV DNA loads in whole blood and PBMCs showed strong correlation [29]. As of April 2023, no other studies have evaluated EBV DNA loads using IU in a cohort of patients with CAEBV. Based on these findings and simplicity of specimen preparation, EBV DNA load ≥ 10,000 IU/mL in whole blood is proposed as the diagnostic cutoff value for CAEBV in this updated guideline. However, high EBV DNA load is also observed in patients with other EBV-associated diseases, such as infectious mononucleosis and EBV-HLH, and occasionally in individuals without any clinical manifestations. Thus, CAEBV cannot be distinguished from other diseases based solely on EBV DNA loads.

#### Identification of EBV-infected cells

EBV-infected cell lineage must be identified for the diagnosis of CAEBV as well as for differential diagnosis of other EBV-associated disorders, because CAEBV is primarily linked to the proliferation of EBV-infected T/NK cells. In situ hybridization (ISH) of the EBV-encoded small RNA (EBER) in a tissue specimen is widely performed for the detection of EBV-infected cells [32]. However, tissue specimens are not always available in clinical settings. EBV-infected cells can be identified in peripheral blood samples by performing real-time PCR on PBMCs fractionated into B, T, and NK cells using an immuno-bead method. Theoretically, larger EBV DNA loads are detected in cell-sorted fractions of an EBV-infected lineage than in unfractionated PBMCs or other uninfected fractions. Therefore, EBV-infected cell lineages can be determined by comparing the EBV DNA loads in each cell-sorted fraction. This method has been used for the diagnosis of CAEBV or other EBV-related disorders [26, 33]. However, owing to the low purity of sorted cells and/or persistent cell-free EBV DNA, false EBV DNA positivity can be detected in some uninfected cell lineages.

Alternatively, EBV-infected cell lineages can be evaluated using fluorescence in situ hybridization (FISH) flow cytometry (flow-FISH) [34–36]. A flow-FISH assay involves flow cytometry with antibody-based staining of surface markers in combination with EBERs, which identifies the EBV-infected cell subset. EBERs are considered the best markers for the detection of EBV-infected cells since they are strongly expressed in all EBV-infected cells [37]. In a previous study conducted using flow-FISH assay, 0.15–67% of the PBMCs of patients with EBV-associated T/NK lymphoproliferative diseases, including nine patients with CAEBV, were EBER-positive T/NK cells [38]. A flow-FISH assay can characterize the details of EBV-infected cell phenotypes; however, its sensitivity for the detection EBV-infected cells is lower than that of real-time PCR assay using fractionated PBMCs as described above. Furthermore, a flow-FISH assay is not available in most diagnostic laboratories.

### Pathological findings

Definitive morphological findings of CAEBV have not been established. Therefore, EBER-ISH studies, which can detect even a few EBV-positive cells in a small tissue specimen, are crucial for the diagnosis of CAEBV. CAEBV is classified as a latency type II infection, which is characterized by the expression of viral antigens (Epstein–Barr virus nuclear antigen 1 and latent membrane proteins 1 and 2). However, immunohistochemical staining rarely detects these antigens in patients with CAEBV. EBV-positive cells often infiltrate the liver, spleen, lymph nodes, and bone marrow; the heart, gastrointestinal system, and muscles are less frequently affected [7]. The lymphocytes that typically infiltrate these organs range in size from small to medium and show no evidence of malignancy. The paracortical hyperplasia and polymorphic proliferation of lymphocytes admixed with other inflammatory cells are frequently observed in the lymph nodes [39, 40]. During disease progression, some patients with CAEBV develop lymphoma or leukemia. Thus, histological examination may reveal a wide range of cytological findings, from reactive appearance to overt leukemia/lymphoma [41].

The lymphocytes are more commonly of T-cell lineage than NK-cell lineage and positive for CD3ɛ and cytotoxic markers such as TIA1 and granzyme B [22, 42]. T-lineage cells are often positive for TCRαβ and CD4, whereas a minority express CD8 and/or TCRγδ [26]. CD56 are positive, particularly in NK-lineage cells. EBV are infected in both T and NK cells in some patients with CAEBV [14].

The clonality of EBV-positive cells has been examined using Southern blot hybridization with a probe that targets EBV terminal repeats [43]. Patients with CAEBV typically exhibit monoclonality; however, some patients have oligoclonal or polyclonal EBV population [41]. Thus, whether CAEBV is a monoclonal lymphoproliferative disorder originating from a single cell remains inconclusive. A reset study showed that driver mutations in a patient with CAEBV were shared by various cell lineages [14]. Therefore, EBV may infect a common lymphoid progenitor and lead to clonal evolution involving multiple cell lineages [18].

## Treatment

**Fig. 1:**
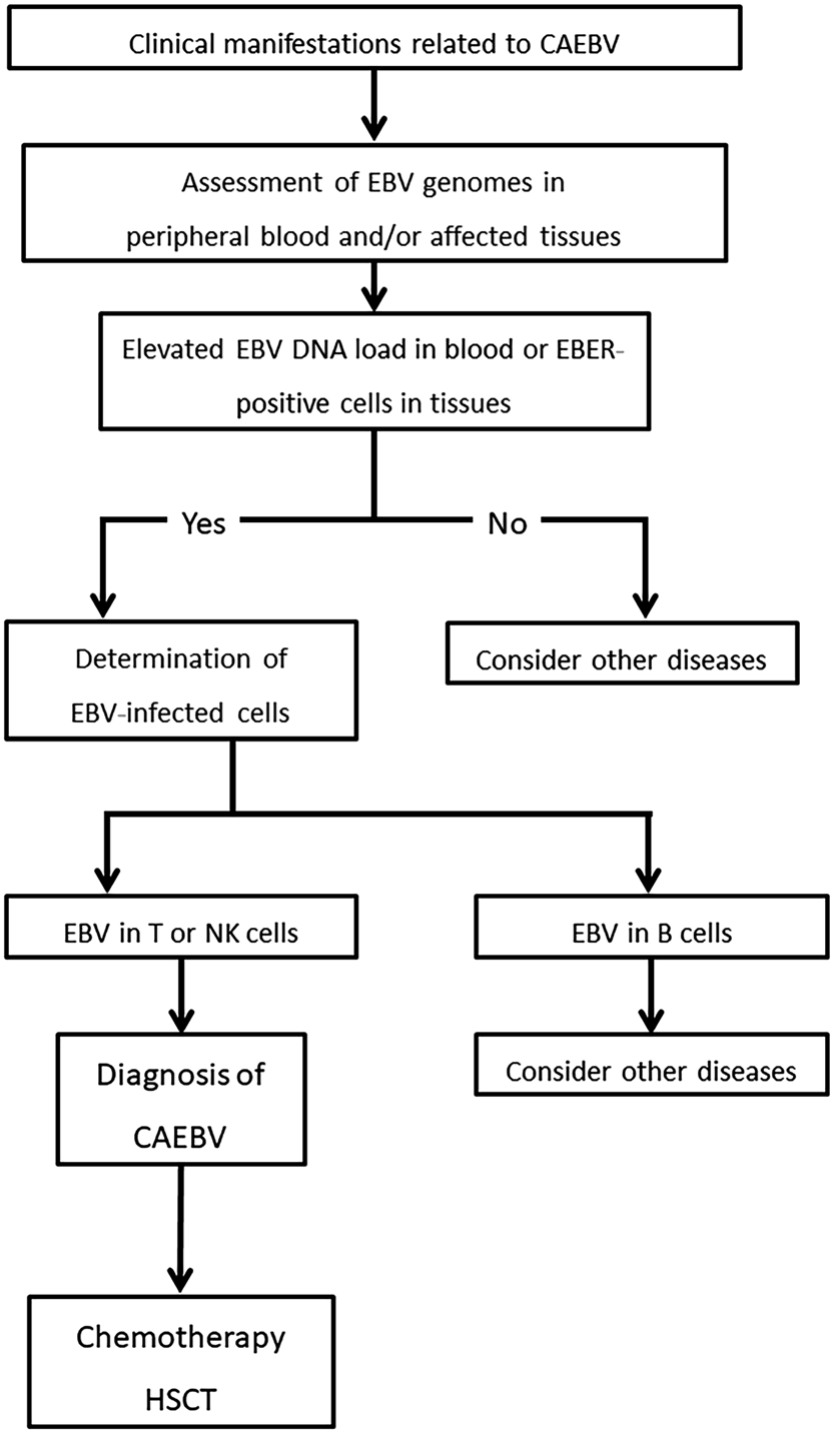
Algorithm for the evaluation and treatment of CAEBV. CAEBV, chronic active EBV disease; EBER, EBV-encoded RNA; HSCT, hematopoietic stem cell transplantation

Various therapeutic options, including administration of anti-viral agents and/or immune-modulatory substances, have been employed for the treatment of CAEBV; however, no standard treatment approach has been established [44]. Currently, hematopoietic stem cell transplantation (HSCT) is considered the only curative treatment for CAEBV. The proposed algorithm for the evaluation and treatment of CAEBV is shown in Fig. 1.

### Chemotherapy

The optimal timing and regimens of chemotherapy for CAEBV are controversial. Chemotherapy can reduce disease activity in CAEBV; however, its effect is usually transient and the rate of complete remission (CR) is insufficient. In a study by Kimura et al., chemotherapy alone induced sustained CR in only 1 of 53 patients with CAEBV [22]. In a recent analysis of 100 cases of CAEBV [21], cyclosporine A, steroids, and etoposide (cooling treatment) were the primary chemotherapy agents administered in 52 cases, whereas cyclophosphamide, doxorubicin, vincristine, and prednisolone (CHOP) were used in 45 cases. The rates of CR after cooling therapy and CHOP were 17% and 13%, respectively. Virological CR, which was defined as CR with a significant decrease in EBV DNA load, was not observed in any patient. Furthermore, the 3-year overall survival (OS) rate for 20 patients treated with chemotherapy alone was 0%. Based on these previous reports, chemotherapy alone was not considered a curative treatment for CAEBV.

Chemotherapy is used to reduce viral load and control disease activity in CAEBV before performing HSCT because the OS rate for patients with inactive disease is significantly higher than that for patients with active disease at the time of HSCT [22]. In addition, chemotherapy could reduce the risk of HSCT-related complications. A combination of immunochemotherapy for the reduction of EBV-infected T/NK cells before proceeding to allogeneic HSCT (three-step strategy, see “Hematopoietic stem cell transplantation”) has been proposed [45, 46]. Chemotherapy may control disease activity in CAEBV and contribute to improving the outcome of HSCT; however, further validation and optimization is required establish its efficacy.

### Hematopoietic stem cell transplantation

The first successful case of allogeneic HSCT for the treatment of CAEBV was reported in 2000 [47]. Since then, various stem cell sources have been used for HSCT for CAEBV [48–53]. HSCT is considered the only curative treatment for CAEBV. In an analysis of 80 cases, the 15-year OS rate for patients with CAEBV treated using HSCT was 60.6%, whereas that for patients who did not undergo HSCT was 25.7% [22]. For the patients who underwent HSCT, age (≥ 15 years), active disease status, and time from onset to HSCT (≥ 30 months) were associated with poor survival. In another study, the 3-year OS rates for patients treated using chemotherapy followed by HSCT and those treated using HSCT only were 65% and 82%, respectively [21]. In a recent study conducted using the Japanese registry database Transplant Registry Unification Management Program (TRUMP), the 3-year OS rate for patients with CAEBV who underwent HSCT (median age, 21 years) was 72.5% [54]. Age (≥ 15 years), disease activity, elevated soluble interleukin-2 receptor level, and conditioning without radiotherapy are independently associated with poor survival. Since CAEBV is rare in Western countries, reports of patients with CAEBV who underwent HSCT are limited. In a previous study by Cohen et al. conducted in the United States, 6 of 19 patients with CAEBV underwent HSCT, and four of the six patients were alive during the observation period [55]. In a recent analysis of patients with T/NK-cell-type CAEBV outside of Asia, those who underwent HSCT had better survival than those who did not; however, relapse was observed in 15 of 44 patients who underwent HSCT [56].

The optimal conditioning and stem cell sources for HSCT for CAEBV have not been established. Kawa et al. retrospectively analyzed 29 patients with CAEBV (median age, 10 years) who underwent HSCT performed after either myeloablative preconditioning (MAC, *n* = 11) or reduced-intensity preconditioning (RIC, *n* = 18) [45]. The 3-year OS rate for patients who underwent RIC was significantly higher than that for patients who underwent MAC (85% vs. 55%). Thus, RIC is a more appropriate preconditioning for HSCT for CAEBV than MAC. In a comparative study of 17 patients with CAEBV who underwent RIC followed by bone marrow transplantation (RIC-BMT) and 15 patients who underwent RIC followed by cord blood transplantation (RIC-CBT), the OS rate for the patients that underwent RIC-BMT was similar to that for patients who underwent RIC-CBT (RIC-BMT, 92.9%; RIC-CBT, 93.3%) [57]. Therefore, unrelated cord blood can be an alternative source for HSCT for CAEBV. However, there are few reports of CBT for the treatment of adult CAEBV cases. Further studies are required to evaluate the superiority of CBT over other stem cell sources for HSCT.

The findings of previous studies indicate that it is crucial to control disease activity in patients with CAEBV at the time of HSCT. The following three-step strategy for the treatment of CAEBV has been proposed [45, 46]:

Step 1: Cooling immunochemotherapy with prednisolone, cyclosporine A, and etoposide

Step 2: Cytoreduction with modified CHOP (cyclophosphamide, pirarubicin, vincristine, and prednisolone) or ESCAP (etoposide, cytosine arabinoside, l-asparaginase, methylprednisolone, and prednisolone)

Step 3: Reconstruction using RIC for HSCT

The 3-year OS rate after planned HSCT for patients with CAEBV treated using this strategy (n = 63) was 87%, whereas that for patients with uncontrolled active disease was 17%.

Owing to the poor prognosis and insufficient efficacy of chemotherapy, HSCT has been recommended as a curative treatment for CAEBV. However, the indication and timing of HSCT should be carefully determined. In some patients, the manifestations of CAEBV are self-limiting and require minimum supportive care [20, 22]. Considering the risk of complications associated with HSCT, sufficient evidence has not yet been accumulated to support the use of HSCT for the treatment of all patients diagnosed with CAEBV. Notably, patients with “active” CAEBV, a condition accompanied by fever, liver dysfunction, vasculitis, or progressive skin lesions, have poorer outcomes after HSCT than patients with inactive disease [54] . Thus, HSCT is recommended when the disease activity of CAEBV is under control. Diagnosis and treatment of CAEBV should be carried out with caution under the guidance of experts of CAEBV.
